# How Does Climate Policy Uncertainty Affect Green Innovation? Evidence from China

**DOI:** 10.3390/ijerph192315745

**Published:** 2022-11-26

**Authors:** Ke Mao, Junxin Huang

**Affiliations:** 1 Business School, Xiangtan University, Xiangtan 411105, China; 2 Business School, Hunan Institute of Technology, Hengyang 421002, China; 3 Chemistry and Environment School, Hunan Institute of Technology, Hengyang 421002, China

**Keywords:** climate policy uncertainty (CPU), green innovation (GI), government subsidies

## Abstract

In response to climate change, governments have adopted various climate policies. However, climate policy uncertainty (CPU) may have important implications for the business sector. Is enterprise green innovation (GI) affected by CPU? This study investigates the impact of CPU on enterprise GI. The China CPU index is created first in this study. It uses panel data from Chinese A-share listed companies in China from 2010 to 2021 to explore the impact of CPU on GI through the fixed effects model, the mediating effects model, and the moderating effects model. The results show that: (1) CPU significantly suppresses GI, according to the findings. (2) CPU inhibits enterprise GI by exacerbating enterprise financing constraints. (3) Government subsidies can mitigate the inhibiting effect of CPU on GI. (4) There is heterogeneity in the negative impact of CPU on enterprise GI, mainly on non-state-owned enterprises. This study suggests several recommendations for coping with CPU in China.

## 1. Introduction

In recent years, serious climate problems have not only caused concern but also posed a threat to people’s health [1,2]. Countries worldwide have adopted relevant climate policies to control greenhouse gas emissions and promote sustainable growth [3]. However, there may be many shortcomings in the development of climate policies by governments. Many governments focus primarily on the cost of policy implementation when developing and implementing climate policies [4], ignoring the uncertainties that may exist on the path to climate policy implementation, i.e., climate policy uncertainty (CPU). For example, the U.S. Government has frequently joined and withdrawn from the Paris agreement in recent years. The uncertainty caused by such policy adjustments often impacts enterprises more critically than the policy itself [5]. Moreover, policy uncertainty shocks often harm enterprises’ production and operations [6], and such impacts are more severe and long-lasting in developing countries [7].

Meanwhile, China, as the world’s largest carbon emitter [8] and energy consumer [9], is working to reduce pollution emissions by promoting enterprise green innovation (GI). GI mainly refers to the innovation activities that promote the development of green technologies, such as energy saving and emission reduction, cleaner production, and renewable energy use [10]. Notably, improving enterprise GI is a critical factor in achieving the goal of green economic development [11,12]. Moreover, enterprise GI is also key to the competitiveness of enterprises [13]. Therefore, exploring whether CPU affects enterprise GI is of practical importance.

Scholars generally agree that environmental regulation is the primary driver of green innovation. For example, Peng et al. (2021) [14] developed a theoretical framework of environmental regulation and GI, arguing that environmental regulation promotes GI behavior by increasing willingness towards GI. Liu and Li (2022) [15] found that China’s pilot policy of carbon emissions trading catalyzes GI in Chinese listed companies with heavy pollution. Liu et al. (2021) [16] used China’s new environmental protection law as a quasi-natural experiment to show that stricter environmental regulations induce more GI in enterprises. Scholars have also investigated the external factors influencing GI in terms of external investment [17,18], financial markets [19,20,21,22,23], political factors [24,25], information disclosure [26,27], and Internet development [28].

Due to the frequent extreme climate problems in recent years, scholars have paid attention to the impact of climate change. Several scholars have investigated the impact of climate change and climate policies on various innovations. For example, Hu et al. (2022) [29] discovered a negative correlation between temperature extremes and GI and a more significant impact on green invention innovation. Since climate change often does not affect enterprises directly but somewhat through climate policy, Pan et al. (2022) [30] used a low-carbon city pilot program as a quasi-natural experiment to explore the impact of climate policy on low-carbon innovation. Closest to our study, Ren et al. (2022) [31] investigated the impact of the U.S. CPU index on the total factor productivity of A-share listed companies in the mining, energy, and manufacturing sectors of China. However, there remains a research gap regarding the impact of CPU on enterprises’ GI. This study aims to examine the impact of Chinese CPU on enterprise GI. First, we work to investigate whether CPU inhibits enterprise GI. Second, we intend to clarify the influencing mechanism of CPU on enterprise GI. Eventually, we analyze whether government subsidies mitigate the negative impact of CPU on enterprise GI.

We organize this paper as follows: Section 2 is a literature review and theoretical hypotheses; Section 3 is an overview of the research methodology; Section 4 describes the data sources and variables; Section 5 contains the empirical results and analysis; Section 6 discusses the research limitations and prospects; and Section 7 lays out the research conclusions and recommendations.

## 2. Literature Review and Research Hypotheses

### 2.1. Literature Review

Initially, the literature focused mainly on measuring the policy uncertainty index. Baker et al. (2016) [32] pioneered a U.S. economic policy uncertainty index based on mainstream news media evaluations using textual analysis methods. Davis (2016) [33] constructed the global economic policy uncertainty index based on the economic policy uncertainty index. The global economic policy uncertainty index covers the weighted average of the economic policy uncertainty indices of 16 major economies around the world (weighted by the GDP of each country). It reflects the trend of global economic policy uncertainty changes more comprehensively.

In recent years, scholars have begun to focus on CPU. Engle et al. (2020) [34] were the first to construct a climate news index by extracting climate-related textual information from *The Wall Street Journal*. Further, Gavriilidis (2021) [35] focused more on climate change-related news. They used eight U.S. newspapers to construct a U.S. CPU index, which laid the foundation for the study of constructing a CPU index.

### 2.2. Research Hypotheses

A higher level of policy uncertainty inhibits enterprise innovation [5]. First, real options theory suggests that an investment opportunity, because there are adjustment costs, can be viewed as an option. Rising uncertainty in the external environment can increase enterprises’ marginal investment cost by raising the option’s value. The irreversible and high-risk characteristics of R&D investment make the enterprise’s innovation activities equivalent to executing a call option, and the investment cost is the option exercise price. Enterprise managers make trade-offs between investments in the current period and future periods. The higher the policy uncertainty, the higher the waiting value of the enterprise’s available funds. This prompts enterprises to reduce their investments [36,37]. Second, policy uncertainty increases enterprises’ capital costs, leading enterprises to reduce production and investment [38]. On the one hand, increased future uncertainty reduces enterprises’ profitability, reducing their financing ability and pushing up their borrowing costs, creating a disincentive for enterprises’ investment decisions [39]. On the other hand, increased policy uncertainty leads banks to reduce credit supply [40], which increases enterprises’ financing constraints and thus discourages enterprise investment. In addition, scholars have found that policy uncertainty in developing countries causes enterprises to postpone investment until reforms are successful [41].

Increased CPU may exacerbate enterprise financing constraints. First, rising policy uncertainty increases the cost of equity and debt financing for enterprises, exacerbating the financing constraint problem for enterprises [42]. Second, increased policy uncertainty can push up banks’ credit risk, prompting them to reduce credit supply [43]. Ultimately, policy uncertainty may lead to lower asset prices, reducing the value of enterprises’ collateral assets and reducing the size of their loanable funds [44]. Furthermore, financing constraints are an important disincentive for enterprises to GI [23,45,46,47].

Government subsidies are an essential tool to correct market failures [48]. On the one hand, government subsidies can mitigate the negative impact of CPU on GI by reducing the problem of financing constraints for enterprises because government subsidies can directly provide enterprises with the funds needed for GI [49]. And. Based on signaling theory, enterprises receiving government subsidies can send more valuable signals about the enterprise to the outside world, reducing the information asymmetry between enterprises and investors and mitigating the inhibitory effect of financing constraints on enterprises’ GI [50]. On the other hand, government subsidies can increase enterprises’ profitability and improve their performance [51], which makes them more desirable for bank credit. The increased available funds for enterprises will reduce their financing constraints and promote their GI. Based on this, we propose the following hypotheses:

**Hypotheses** **(H1):** *Increased CPU inhibits enterprise GI*.

**Hypotheses** **(H2):** *CPU inhibits enterprise GI by exacerbating enterprise financing constraints*.

**Hypotheses** **(H3):** *Government subsidies can mitigate the inhibiting effect of CPU on GI*.

## 3. Empirical Model and Methodology

### 3.1. Fixed Effects Model

This study empirically examined the impact of CPU on enterprise GI. Referring to Irfan et al., (2022) [19], we consider different types of GI as dependent variables. We constructed the following econometric model with a lagged one-period CPU index as the core independent variable. Since enterprises usually base their investment plans for the coming year on the previous year’s external environment and business conditions, enterprise GI requires a high degree of continuity. Therefore, when enterprise managers notice the adjustment of climate policy in the current year, they can only change their GI decisions in the following year as well. Based on previous theoretical analysis (Hypothesis 1), we constructed a fixed-effects model as follows:

(1) $$GI_{i,t}=\alpha_{0}+\alpha_{1}CPU_{t-1}+\sum control_{i,t}+\mu_{i}+\varepsilon_{i,t}$$

where the subscripts *i* and *t* subscripts represent enterprise *i* and year *t*; *CPU_t−1_* indicates the CPU index in year *t* − 1; $GI_{i,t}$ represents the level of GI of enterprise *i* in year *t*. Meanwhile, to mitigate possible bias caused by other enterprise-level factors, we control for a series of control variables. In addition, to mitigate the effect of individual heterogeneity on enterprise GI, we also considered individual effects $\mu_{i}$ to control for inherent enterprise characteristics that do not vary over time and are difficult to observe. $\varepsilon_{i,t}$ represents the unobservable error term.

### 3.2. Mediating Effect Model

Furthermore, to test whether CPU affects the enterprise’s GI through the enterprise’s financing constraints (Hypothesis 2). Refer to [52,53,54], we construct the mediating effects model as follows:

(2) $$SA_{i,t}=\beta_{0}+\beta_{1}CPU_{t-1}+\sum control_{i,t}+\mu_{i}+\varepsilon_{i,t}$$

(3) $$GI_{i,t}=\gamma_{0}+\gamma_{1}CPU_{t-1}+\gamma_{2}SA_{i,t}+\overset{}{\underset{}{\sum }}control_{i,t}+\mu_{i}+\varepsilon_{i,t}$$

where $SA_{i,t}$ represents enterprise financing constraints in enterprise i in year t. Our study concentrates on the coefficients $\beta_{1}$ and $\gamma_{2}$. If the coefficients of both $\beta_{1}$ and $\gamma_{2}$ are significant, there is a mediating effect. Otherwise, there is no mediating effect.

### 3.3. Moderating Effect Model

To identify the moderating effect of government subsidies (Hypothesis 3). Referring to the literature [55], we construct a moderating effect model as follows. We use the interaction term $CPU_{t-1}\times subsidies_{i,t}$ to determine the moderating effect of government subsidies.

(4) $$GI_{i,t}=\omega_{0}+\omega_{1}CPU_{t-1}+\omega_{2}CPU_{t-1}\times subsidies_{i,t}+\omega_{3}subsidies_{i,t}+\sum control_{i,t}+\mu_{i}+\varepsilon_{i,t}$$

where $subsidies_{i,t}$ represents the government subsidies received by the enterprise i in year t. The interaction term coefficient $\omega_{2}$ represents the moderating effect of government subsidies on the enterprise’s GI. If the coefficient $\omega_{2}$ is significant, there is a moderating effect. Otherwise, there is no moderating effect.

## 4. Definitions of Variable and Data Sources

### 4.1. Independent Variable

Referring to Gavriilidis (2021) [35], we constructed the China CPU index. We selected keywords from three levels: climate, policy, and uncertainty, and calculated the China CPU index by counting the word frequency of keyword occurrences, which can more objectively, comprehensively, and accurately reflect changes in China’s climate policy. Table 1 shows the retrieved keywords and their English translations.

We identified the articles about China’s CPU published in newspapers each month and divided the number of identified articles by the total number of articles published that month. Then, we calculated the monthly average of the two newspapers and normalized it to obtain the monthly China CPU index. Eventually, we obtained the CPU index for China and took the logarithmic value, which is denoted as *lnCPU*.

### 4.2. Dependent Variable

Referring to the literature [20,56], we measured the GI level by taking the natural logarithm of the number of green invention patents granted (IPG) plus one and the natural logarithm of the number of green utility model patents granted (UMPG) plus one, respectively, which are denoted as *lnIPG* and *lnUMPG*.

### 4.3. Mediating Variable

For Hypothesis 2, we focused on the enterprise financing constraint as the mediating variable. Referring to Wu and Huang (2022) [2], we used the KZ index to measure the level of enterprise financing constraints, denoted as KZ. The larger the KZ index, the higher the degree of enterprise financing constraints.

### 4.4. Moderating Variable

For Hypothesis 3, we chose government subsidies as the moderating variable. Referring to the literature of Xu et al. (2018) [57], we measured the natural logarithm of government subsidies, denoted as Subsidies.

### 4.5. Controlled Variables

Referring to the literature [58], we selected a vector of enterprise-level control variables. Enterprise size (*lnSize*) is defined as the natural logarithm of the enterprise’s total assets; enterprise leverage (*Lev*) is expressed as the ratio of total liabilities to total assets; enterprise investment opportunity (*TobinQ*) is expressed as the ratio of the sum of stock market value, and total debt to total assets; enterprise profitability (*Roa*) is expressed as the ratio of net profit to total assets; and enterprise age (*lnAge*) was calculated from the year of enterprise establishment and took the logarithmic value. Table 2 displays descriptive statistics of the variables. 

Besides, the relevant economic and financial terms in the text are explained as shown in Table A1.

### 4.6. Statistical Characteristics

Table 2 provides descriptive statistics of the sample in this study. The mean value of lnIPG is 0.141, and the mean value of *lnUMPG* is 0.251. Considering the innovation stock, which implies that the number of green patents granted in China is low, combined with the types of green patents granted by enterprises, the number of green IPG is less than the number of green IMPG by Chinese enterprises.

### 4.7. Data Sources

From 2010 to 2021, we selected a sample of A-share listed companies in Shanghai and Shenzhen. Referring to Ren et al. (2022) [31], the initial sample was processed as follows: (1) excluded the sample of financial listed companies; (2) excluded the sample treated by ST and *ST during the sample period; (3) excluded the sample of the year of IPO; (4) excluded the abnormal companies with negative net assets and financial indicator samples; (5) excluded samples with missing key variables; and eventually we obtained 25,513 observed samples. Among them, the data to measure the GI of enterprises were obtained from the China Research Data Service Platform (CNRDS). The financial data of listed companies was obtained from the Cathay Capital (CSMAR) database. 

## 5. Empirical Results

### 5.1. Baseline Regression

This paper first analyzes the relationship between CPU and GI, i.e., to test Hypothesis 1. Table 3 reports the regression results of CPU affecting GI. Among them, columns (1)–(3) are the regression results with *lnIPA* as the dependent variable, and columns (4)–(6) are the regression results with *lnUMPA* as the dependent variable. Specifically, column (1) regresses only the key independent variable *CPU* without adding control variables, and the results show that the coefficient of *CPU* is significantly negative. Column (2) adds all control variables, the explanatory strength of the model increases significantly, and the results are more precise. Moreover, the coefficient of *CPU* remains significantly negative. Furthermore, column (3) adds individual fixed effects. The regression coefficient is −0.033, which is significant at the 1% level. Similarly, the regression results for columns (4)–(6) show that the coefficients of *CPU* are also all significantly negative at the 1% level. Empirical analysis results show that Hypothesis 1 was tested. 

In addition, the regression results for the control variables show that the regression coefficients of *lnSize* and *lnAge* are significantly positive. It indicates that the larger the enterprise size is, the higher the level of GI for the enterprise. The higher the number of enterprise years, the higher the level of enterprise GI. The regression coefficients of enterprise *Roa*, *TobinQ*, and *Lev* are insignificant.

### 5.2. Robustness Tests

To ensure the reliability of the study results, we also performed extensive robustness tests. The results are shown in Table 4. First, we added a series of macro variables, such as GDP growth rate, M2 growth rate, and CPI growth rate, to the control variables. As can be seen from columns (1) and (2), the regression coefficient of CPU is still significantly negative at the 1% level. The baseline regression results remain robust. Second, we replaced the independent variable. Our study on China’s CPU mainly used the China CPU index to reduce measurement bias. Referring to existing studies, we used the U.S. CPU index constructed by Gavriilidis (2021) [35] to replace the independent variable for further robustness tests. The regression results are shown in columns (3) and (4), and the coefficient of CPU is significantly negative at the 5% level. The benchmark regression results remain robust. Third, we replaced the dependent variables. To further ensure the robustness of the benchmark regression results, we used the green patent application instead of the green patent grant for the empirical test. Columns (5) and (6) report the results after replacing the dependent variables, which are still robust. Hence, the conclusion of Hypothesis 1 is robust.

### 5.3. Mediating Effects

To test Hypothesis 2, i.e., the mechanism of action of CPU influence on GI, we conducted a mediating effect test using Equations (2) and (3), and the test results are shown in Table 5. The regression coefficient of the explanatory variable *lnCPU* on the mediating variable KZ in column (1) is significantly positive at the 1% level, indicating that the increase in CPU exacerbates the financing constraint problem for enterprises. Columns (2) and (3) report the regression results for *lnIPG* and *lnUMPG* as dependent variables, respectively. The results show that the regression coefficients of both CPU and the mediating variable KZ are significantly negative, indicating that enterprise financial constraints play a partial mediating effect between CPU and GI. Thus, research Hypothesis 2 was verified.

### 5.4. Moderating Effects

The results of the previous study show that the rise in CPU increases enterprises’ financing dilemma, which reduces the GI. If the climate policy is frequently adjusted, the uncertainty of the development direction of enterprises and the increase in business risks reduces the willingness of entrepreneurs to carry out innovation. However, government subsidies can increase the confidence of enterprises facing CPU, motivate their R&D and innovation activities by increasing their R&D funds, reduce the risk of R&D and technology spillover, and alleviate the financing constraint problem, thus lowering the uncertainty of the external environment.

To test Hypothesis 3, i.e., the moderating effect of government subsidies on CPU impact GI, we used equation (4) to investigate the moderate effect. The results are shown in Table 6. The interaction term between CPU and government subsidies in column (1) is significantly positive at the 1% level, indicating that government subsidies weaken the negative effect of CPU on GI. Column (2) adds individual fixed effects to column (1). The results show that the interaction term between CPU and government subsidies still has a significantly positive effect on GI, further verifying the positive moderating effect of government subsidies. In addition, columns (3) and (4) take the green utility model patent grant as the dependent variable, and the interaction term between CPU and government subsidies remains significantly positive, further ensuring the robustness of the study findings. Thus, Hypothesis 3 is confirmed.

### 5.5. Heterogeneity Analysis

Considering that the inhibitory effect of CPU on GI may be affected by the heterogeneity of different enterprises, we divided the sample of enterprises into state-owned enterprises (SOE) and non-state-owned enterprises (non-SOE) for group regression analysis. The results are shown in Table 7. Columns (1) and (3) report the regression results for the SOE sample. Although the regression coefficient of CPU is still negative, it is not significant. This indicates that CPU has no significant effect on the GI of SOEs. Columns (2) and (4) report the regression results for the non-SOE sample. As can be seen, unlike the empirical results for the SOE sample, the regression coefficient for CPU is significantly negative at the 1% level. The results indicate that CPU only has a significant inhibitory effect on non-SOEs.

The heterogeneous impact of CPU on GI across ownership may be due to the differences between SOEs and non-SOEs not only in terms of resource endowment, goals, and values but also in terms of the implementation of national policies and the assumption of environmental responsibilities. SOEs occupy a unique position in China and usually have more stringent environmental protection systems and are more concerned with long-term enterprise development and environmental and economic benefits to society. The importance of GI is more evident in SOEs than in non-SOEs. In addition, SOEs have a natural advantage in obtaining external financing and have fewer financing constraints. The impact of CPU on SOEs’ financing constraints is even less. Therefore, there is a significant difference in the impact of CPU on GI in heterogeneous enterprises.

## 6. Research limitations and Prospects

Certain research shortcomings in this paper deserve further exploration in future studies. First, due to data availability, we mainly used data from listed companies in China and no data from non-listed companies. Future research could include a broader sample of enterprises to study. Second, our study only examined the impact of direct government subsidies on enterprise GI. Future research needs to more comprehensively consider the heterogeneous impact of different forms of government subsidies, which may become an important direction for future research. Eventually, our study only considers the adverse effects of government subsidies that can mitigate CPU. Future research could explore more influencing factors to mitigate the harmful effects of CPU and enrich and improve the existing studies.

## 7. Conclusions

The adverse effect of CPU has caused widespread concern in recent years. We constructed an index of CPU in China based on two major Chinese newspapers, adopting the panel regression model, the mediating effect model, and the moderating effect model. We investigated the impact of CPU on enterprise GI using data from A-share-listed Chinese companies from 2010 to 2021. It was found that CPU has a dampening effect on enterprise GI, i.e., the higher the CPU, the lower the enterprise GI. The findings were subjected to a series of robustness tests to ensure reliability. Second, the financing constraint faced by enterprises is a mediating path through which uncertainty in enterprises’ climate policies affects their GI. A rise in CPU exacerbates enterprises’ financial constraints, which in turn inhibits enterprises’ GI activities. Third, government subsidies can significantly mitigate the inhibitory effect of CPU on enterprises’ GI. Ultimately, the negative effect of CPU differs across heterogeneous enterprises, with CPU mainly hurting the GI of non-SOEs and having no significant effect on SOEs.

The main contributions of this paper are the following: First, we extended the literature on the impact of policy uncertainty on enterprise innovation. Most previous studies have explored the impact of economic and trade policy uncertainty on enterprises’ traditional innovations [42,59,60,61]. There needs to be more literature on policy uncertainty in GI. Unlike traditional innovation, GI is an innovation activity that aims to protect the environment [62]. Only Xu and Yang (2021) [63] have investigated the impact of economic policy uncertainty on GI in enterprises. In contrast to these studies, our study examines the impact of CPU on enterprise GI in China.

Second, our study extends the literature on the construction of policy uncertainty indices. Several recent studies have investigated the impact of CPU in the United States [64,65,66]. However, these studies ignore the fact that there are significant differences in economic, social, and climate policies across countries, and the use of the U.S. CPU index as a proxy variable for national uncertainty may lead to problems with biased findings. Drawing on the U.S. CPU index developed by Gavriilidis (2021) [35], we constructed an index of CPU for China.

Eventually, our study extends the literature on how government subsidies affect enterprise innovation. Extensive literature has focused on the direct impact of government subsidies on enterprise innovation [57,67,68,69,70]. However, little attention has been paid to whether government subsidies play an essential role in the relationship between CPU and enterprise GI. This study uses a moderating effects model to show that government subsidies mitigate the negative effects of CPU on enterprise GI.

Based on the above findings, we can obtain the following policy insights: (1) Given that CPU can exacerbate the problem of enterprise financing constraints and inhibit the level of enterprise GI, the government should pay attention to the adverse effects of CPU on enterprises, pay attention to the frequency of policy adjustments when making climate policy adjustments, and keep the policies as long-term and consistent as possible. At the same time, the government should strengthen communication with the business sector to help them prepare well in advance to cope with CPU and reduce its negative impact of CPU. (2) Government subsidies can effectively mitigate the inhibiting effect of CPU on GI. Therefore, the government should implement more preferential policies on enterprise R&D innovation to, on the one hand, reduce enterprise financing constraints and increase available funds for enterprise GI, and on the other hand, pay attention to the effectiveness of government incentives and target promoting enterprise GI to achieve sustainable economic growth. (3) CPU has a greater inhibitory effect on private enterprises’ GI, and these enterprises are generally less capable of financing and innovation. Therefore, when encouraging GI in enterprises, the government should pay attention to the differences in ownership and formulate targeted encouragement and support policies.

## Figures and Tables

**Table 1:** Related Keywords.

Criteria	English	Chinese
Uncertainty	Uncertainty/uncertain	不确定性/不确定
	Volatile	动荡
	Unstable/unclear	不稳/不明朗
	Unpredictable	难以预料
Climate change	Carbon dioxide	二氧化碳
	Climate	气候
	Climate risk	气候风险
	Greenhouse gas emissions	温室气体排放
	Greenhouse	温室
	CO_2_	CO_2_
	Emissions	排放
	Global warming	全球变暖
	Climate change	气候变化
	Green energy	绿色能源
	Renewable energy	可再生能源
	Environmental	环境
Policy	Policy/measures	政策/措施
	Politics	执政
	Government/authority	政府/中央
	President	国家主席
	Prime minister	总理
	Reform	改革
	Regulation	监管
	Environmental Protection Administration	环保局

**Table 2:** Descriptive statistics.

Variables Type	Variables	Full Name	Obs.	Mean	Std. Dev.	Min	Max
Dependent variable	*lnCPU*	Climate policy uncertainty index	12	88.070	23.430	66.945	140.074
Independent variable	*lnIPG*	The number of green invention patents granted	25,513	0.141	0.456	0.000	5.043
	*lnUMPG*	The number of green utility model patents granted	25,513	0.251	0.612	0.000	5.844
Mediating variable	*KZ*	Financing constraints	25,513	−3.742	0.267	−5.600	−0.271
Moderating variable	*Subsidies*	Government subsidies	25,513	0.014	0.060	0.000	8.380
Controlled variables	*lnSize*	Enterprise size	25,513	22.081	1.368	13.076	28.636
	*lnAge*	Enterprise age	25,513	9.533	7.207	−1.000	29.000
	*Roa*	Enterprise profitability	25,513	0.039	0.798	−48.316	108.366
	*TobinQ*	Enterprise investment opportunity	25,513	2.356	13.447	0.674	52.705
	*Lev*	Enterprise leverage	25,513	0.445	0.644	−0.195	63.971

**Table 3:** Effect of CPU on GI.

Variable	*lnIPG*			*lnUMPG*		
	(1)	(2)	(3)	(4)	(5)	(6)
*lnCPU*	−0.051 ***	−0.039 ***	−0.033 ***	−0.208 ***	−0.200 ***	−0.037 ***
	(0.012)	(0.012)	(0.008)	(0.016)	(0.016)	(0.011)
*lnSize*		0.109 ***	0.019 ***		0.118 ***	0.018 **
		(0.003)	(0.005)		(0.004)	(0.007)
*lnAge*		0.007 ***	0.009 ***		−0.014 ***	0.011 ***
		(0.000)	(0.001)		(0.001)	(0.001)
*Roa*		−0.108 ***	−0.022		−0.027	0.001
		(0.028)	(0.021)		(0.037)	(0.028)
*TobinQ*		0.112 ***	0.005		0.072 ***	−0.002
		(0.009)	(0.009)		(0.012)	(0.011)
*Lev*		−0.059 ***	−0.029		0.154 ***	0.086 **
		(0.021)	(0.026)		(0.029)	(0.034)
*Cons*	−0.088 *	−2.585 ***	−0.221 *	−0.676 ***	−3.229 ***	−0.436 ***
	(0.053)	(0.086)	(0.124)	(0.070)	(0.116)	(0.163)
*N*	25,513	25,513	25,513	25,513	25,513	25,513
*Enterprise FE*	NO	NO	YES	NO	NO	YES
*Adj. R^2^*	0.010	0.151	0.253	0.017	0.164	0.259

Note: robustness standard errors are in parentheses; ***, **, * denote passing the test at 1%, 5%, and 10% significance levels, respectively.

**Table 4:** Robustness test.

Variable	*lnIPG*	*lnUMPG*	*lnIPG*	*lnUMPG*	*lnIPA*	*lnUMPA*
	(1)	(2)	(3)	(4)	(5)	(6)
*lnCPU*	−0.038 ***	−0.056 ***			−0.003 ***	−0.004 ***
	(0.013)	(0.017)			(0.001)	(0.001)
*lnACPU*			−0.029 **	−0.066 ***		
			(0.011)	(0.013)		
*lnSize*	0.019 ***	0.011	0.020 ***	0.012	0.043 ***	0.012
	(0.007)	(0.009)	(0.007)	(0.009)	(0.011)	(0.010)
*lnAge*	0.007 **	0.006	0.010 ***	0.011 ***	0.017 ***	0.011 ***
	(0.003)	(0.004)	(0.001)	(0.002)	(0.002)	(0.002)
*Roa*	−0.023	0.002	−0.023	0.006	0.040 *	0.039 **
	(0.019)	(0.020)	(0.019)	(0.020)	(0.023)	(0.020)
*TobinQ*	0.007	−0.024 **	0.008	−0.021 **	0.023 *	−0.003
	(0.010)	(0.012)	(0.009)	(0.011)	(0.013)	(0.012)
*Lev*	0.034	0.105 ***	0.026	0.103 ***	0.051	0.045
	(0.029)	(0.032)	(0.029)	(0.032)	(0.037)	(0.034)
*GDP*	0.040	−0.852 **				
	(0.297)	(0.378)				
*m2*	−0.149	0.277				
	(0.166)	(0.221)				
*Cpi*	0.039	0.516				
	(0.255)	(0.355)				
*Cons*	−0.205	−0.098	−0.401 ***	−0.143	−0.986 ***	−0.453 ***
	(0.128)	(0.168)	(0.122)	(0.160)	(0.173)	(0.167)
*N*	25,513	25,513	25,513	25,513	25,513	25,513
*Enterprise FE*	YES	YES	YES	YES	YES	YES
*Adj. R^2^*	0.153	0.156	0.154	0.160	0.139	0.157

Note: robustness standard errors are in parentheses, ***, **, * denote passing the test at 1%, 5%, and 10% significance levels, respectively.

**Table 5:** Mediating effects test.

Variable	(1)	(2)	(3)
	KZ	*lnIPG*	*lnUMPG*
*lnCPU*	0.018 ***	−0.048 ***	−0.040 ***
	(0.002)	(0.012)	(0.014)
*KZ*		−0.350 ***	−0.141 *
		(0.087)	(0.073)
*lnSize*	−0.041 ***	0.032 ***	0.018*
	(0.007)	(0.009)	(0.010)
*lnAge*	−0.035	0.021 ***	0.011 ***
	(0.001)	(0.004)	(0.003)
*Roa*	0.029	−0.032 *	−0.002
	(0.024)	(0.019)	(0.020)
*TobinQ*	0.040 ***	−0.012	−0.024 **
	(0.010)	(0.009)	(0.011)
*Lev*	0.044	0.020	0.099 ***
	(0.032)	(0.031)	(0.033)
*Cons*	−2.639 ***	0.768 ***	0.444 *
	(0.141)	(0.223)	(0.233)
*N*	25,513	25,513	25,513
*Enterprise FE*	YES	YES	YES
*Adj. R^2^*	0.765	0.147	0.159

Note: robustness standard errors are in parentheses, ***, **, * denote passing the test at 1%, 5%, and 10% significance levels, respectively.

**Table 6:** Moderating effects test.

Variable	(1)	(1)	(2)	(2)
	*lnIPG*	*lnIPG*	*lnUMPG*	*lnUMPG*
*lnCPU*	−0.028 ***	−0.039 ***	−0.014 ***	−0.038 ***
	(0.009)	(0.012)	(0.02)	(0.014)
lnCPU *subsidy	0.131 ***	0.190 ***	0.378 ***	0.140 ***
	(0.030)	(0.031)	(0.082)	(0.045)
*subsidy*	−0.368	0.889	−1.423	0.047
	(1.326)	(1.347)	(1.690)	(1.423)
*lnSize*	0.064 ***	0.018 **	0.067 ***	0.012
	(0.004)	(0.007)	(0.005)	(0.009)
*lnAge*	−0.001 *	0.009 ***	−0.006 ***	0.006 ***
	(0.001)	(0.002)	(0.001)	(0.002)
*Roa*	−0.056 ***	−0.022	−0.029	0.001
	(0.020)	(0.019)	(0.026)	(0.020)
*TobinQ*	0.034 ***	0.002	0.008	−0.019 *
	(0.008)	(0.009)	(0.010)	(0.011)
*Lev*	−0.016	0.035	0.079 ***	0.104 ***
	(0.022)	(0.029)	(0.029)	(0.032)
*Cons*	−1.162 ***	−0.169	−1.147 ***	0.065
	(0.096)	(0.171)	(0.127)	(0.206)
*N*	25,513	25,513	25,513	25,513
*Enterprise FE*	NO	YES	NO	YES
*Adj. R^2^*	0.153	0.153	0.160	0.160

Note: robustness standard errors are in parentheses, ***, **, * denote passing the test at 1%, 5%, and 10% significance levels, respectively.

**Table 7:** Heterogeneity analysis.

Variable	(1) SOE	(2) Non-SOE	(3) SOE	(4) Non-SOE
	*lnIPG*	*lnIPG*	*lnUMPG*	*lnUMPG*
*lnCPU*	−0.015	−0.043 ***	−0.007	−0.075 ***
	(0.013)	(0.011)	(0.016)	(0.015)
*lnSize*	0.021 **	0.022 ***	−0.002	0.033 ***
	(0.009)	(0.007)	(0.011)	(0.009)
*lnAge*	0.012***	0.006 ***	0.014 ***	0.007 ***
	(0.001)	(0.001)	(0.002)	(0.002)
*Roa*	−0.063	−0.013	0.037	−0.016
	(0.042)	(0.024)	(0.052)	(0.033)
*TobinQ*	0.031 *	−0.001	−0.024	0.016
	(0.016)	(0.011)	(0.020)	(0.015)
*Lev*	0.031	0.037	0.135 **	0.065
	(0.048)	(0.031)	(0.059)	(0.042)
*Cons*	−0.461 **	−0.213	0.119	−0.872 ***
	(0.212)	(0.157)	(0.260)	(0.216)
*N*	YES	YES	YES	YES
*Enterprise FE*	9831	15,682	9831	15,682
*Adj. R^2^*	0.107	0.184	0.118	0.187

Note: robustness standard errors are in parentheses, ***, **, * denote passing the test at 1%, 5%, and 10% significance levels, respectively.

## Data Availability

The data in this study are available on request from the authors.

## Appendix A

**Table A1:** Terms statement.

Term	Statement
Financing constraints	The financing constraint refers to the ease of access to capital relative to the investment opportunities of the firm.
Government subsidies	Government subsidies are non-reimbursable financial support given by local governments to stimulate innovative activities of local enterprises according to economic or policy guidelines at different times
*GDP*	GDP is the gross domestic product.
*m2*	m2 is the money in circulation generated through commercial banks
*cpi*	cpi refers to the consumer price index, reflecting the trend and degree of price changes of consumer goods and services purchased by urban and rural residents in a certain period of time, and is the result of a comprehensive summary calculation of urban consumer price index and rural consumer price index.
